# Intercostal hemangiomas coexisting with multiple hepatic hemangiomas: Clinical and imaging findings in a single case report with a review of the literature

**DOI:** 10.1097/MD.0000000000037261

**Published:** 2024-02-23

**Authors:** Xiangui Li, Xing Wen, Lin Yang

**Affiliations:** a Department of Radiology, Ziyang People’s Hospital, Ziyang, Sichuan, China.

**Keywords:** case report, clinical, imaging, intercostal hemangioma, multiple hepatic hemangiomas

## Abstract

**Rationale:**

Intercostal hemangioma (IH) is an extremely rare disease, with only 18 cases reported in the past 30 years. Herein, we report the first case of IH coexisting with multiple hepatic hemangiomas, which recurred 32 months after surgery with rib erosion. IHs are invasive and difficult to distinguish from other intercostal tumors on imaging. To date, there have been no review articles on the imaging findings of IHs. We hope that this article will help clinicians improve their ability to diagnose and treat IH.

**Patients concerns:**

A 58-year-old male came to our hospital with gastrointestinal disease. Chest tumors were accidentally discovered on routine chest computed tomography (CT). The patient had no chest symptoms. The patient also had multiple liver tumors that had been present for 2 years but with no remarkable changes.

**Diagnosis:**

Plain chest CT revealed 2 adjacent masses protruding from the left chest wall into the thoracic cavity. Neurogenic tumors or hamartomas were suspected on enhanced CT scans. Abdominal contrast-enhanced computed tomography scan indicated multiple liver tumors as MMHs, which was consistent with the 2 previous Doppler ultrasound findings.

**Interventions:**

Surgeons removed the chest tumors by video-assisted thoracoscopic surgery. No treatment was provided for the MMHs.

**Outcomes:**

Two tumors of the chest wall were diagnosed as the IHs. There were no significant changes in the hepatic tumors after 32 months of follow-up. Unfortunately, the IH recurred, and the left 5th rib was slightly eroded.

**Lessons:**

It is necessary to include IHs as a potential differential diagnosis for chest wall tumors because early clinical intervention can prevent tumor growth and damage to adjacent structures. The imaging findings of IH show special characteristics. Preoperative imaging evaluation and diagnosis of IH are helpful for safe and effective surgery. Because of the high recurrence rate, complete surgical resection of IH with a sufficient tumor-free margin is recommended. It should be noted that the ribs should also be removed when the surrounding ribs are suspected to have been violated.

## 1. Introduction

Intercostal hemangioma (IH) is easily forgotten in the differential diagnosis of intercostal tumors. Patients often visit hospital with a painless mass in their chest. The preoperative imaging diagnosis of IH is difficult, and it usually needs to be confirmed by pathology after surgical resection. Herein, we report a case of IH coexisting with multiple hepatic hemangiomas (MHHs) that recurred 32 months after surgery with rib erosion. Our patient had 2 IHs. Computed tomography (CT) scans failed to provide a definitive diagnosis. The patient underwent video-assisted thoracoscopic surgery (VATS). Intercostal muscle venous hemangiomas (VHs) were finally diagnosed. In addition, multiple liver tumors were diagnosed as MHHs by multiple Doppler ultrasound examinations, CT scans, and follow-up observations. Combined with this case, we reviewed the clinical and imaging findings of 18 cases of IH reported in the English literature over the past 30 years. To the best of our knowledge, this is the first case of its type to be reported and there is no detailed review of the imaging findings of IH.

## 2. Case presentation

A 58-year-old man visited our hospital for treatment with upper abdominal discomfort. He had pulmonary tuberculosis 30 years ago and the symptoms disappeared after treatment. Two years ago, abdominal Doppler ultrasound examination suggested multiple hepatic tumors and MHHs were considered. The patient had no symptoms of chest discomfort and his physical examination showed no abnormality. His granddaughter had a congenital hemangioma on her forehead.

**Figure 1.**
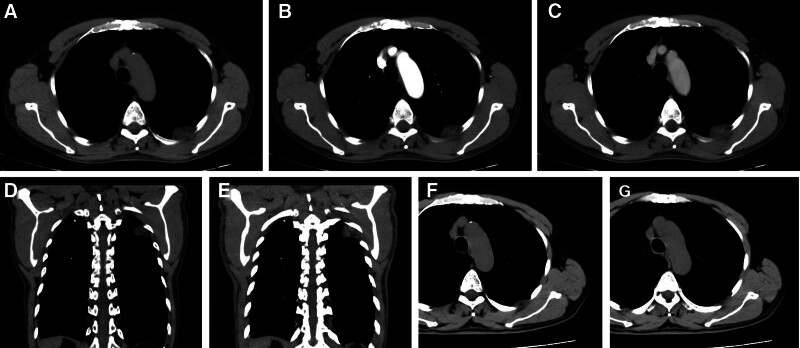
Two adjacent tumors on the chest wall. The normal intercostal muscle structure was had been destroyed (A); nodular/tubular and central enhancement (B, D); contrast agent filling (C, E); recurrence of intercostal hemangioma (F), and erosion of the adjacent rib (G).

Gastroscopy revealed esophagitis, erosive gastritis and duodenitis. Routine plain chest CT scan images revealed 2 adjacent tumors on the left chest wall, with slightly uneven density and sizes of 1.9 × 1.3 cm and 1.1 × 0.9 cm, respectively. The tumors were located in the 5th intercostal region, protruding into the thoracic cavity, surrounded by a “capsule,” and attached to the chest wall with a broad base; the normal intercostal muscle structure was destroyed (Fig. 1A). The CT values of tumors ranged from −10HU to 10HU with an average of 1HU. Plain abdominal CT revealed multiple slightly low-density tumors in the liver. Abdominal Doppler ultrasound revealed multiple hepatic tumors, which were considered to be MHHs.

**Figure 2.**
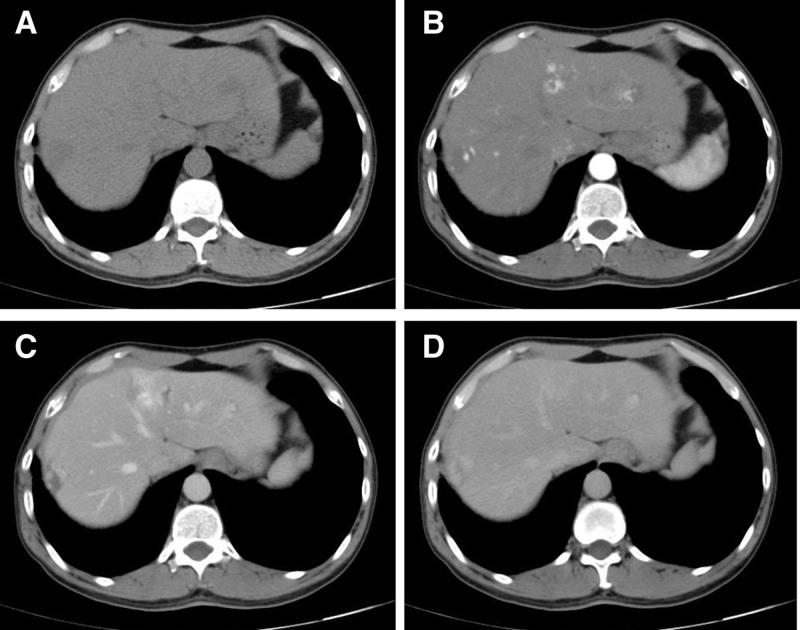
The typical enhancement pattern of hepatic hemangiomas. Plain scan (A); arterial phase (B); vein phase (C); and delayed phase (D).

According to contrast-enhanced computed tomography (CECT) images of chest, in the arterial phase (Fig. 1B, 1D), the 2 tumors presented with nodular/tubular and central enhancement (CT values ranged from 0HU to 34HU, with an average of 16HU), and in the delayed phase (Fig. 1C, 1E), the contrast agent showed further enhancement of filling (CT values ranged from 0HU to 40HU, with an average of 29HU). The CECT diagnosis suggested that these may be neurogenic tumors or hamartomas, even though they exhibited “fast in, slow out,” progressive filling and vascular-like structural enhancement. This may be due to the fact that hemangiomas are rarely detected in this region. In addition, the liver tumors exhibited an intensification mode of fast advance and slow exit and centripetal filling (Fig. 2). These tumors were considered as MHHs because of the typical enhanced patterns for hepatic hemangiomas.

**Figure 3.**
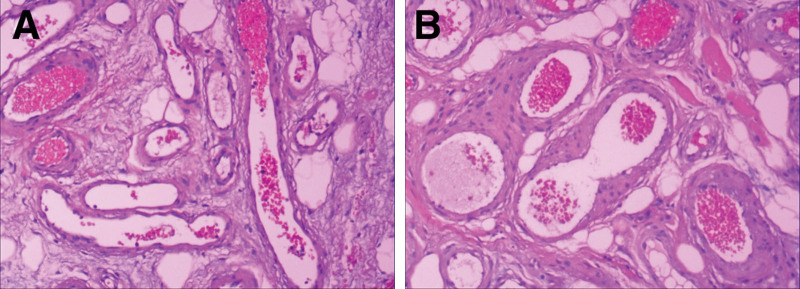
Hematoxylin and eosin staining of intercostal tumors (400×). The walls of blood vessels were made of smooth muscle of varying thickness, and red blood cells are filled or scattered in some vascular lumens; the lumens varied in size, they were separated by fibrous connective tissue, and adipocytes were seen (A, B).

The surgeon performed VATS exploration of the patient and found that the tumors were surrounded by parietal pleura with clear boundaries. When the parietal pleura was opened, 2 tumors were visible inside. The ribs were intact. The surgeon considered these to be benign tumors, so they were completely removed from the chest wall without extending the resection. Pathological staining showed that the walls of the blood vessels were made of smooth muscle of varying thickness. The lumens varied in size and were separated by fibrous connective tissue. Adipocytes were evident. The red blood cells were filled or scattered in some vascular lumens. Analysis confirmed that these were VHs (Fig. 3A, 3B) originating from the intercostal muscle. The patient presented with multiple hepatic masses without any symptoms of hepatic discomfort. The patient’s 2 Doppler ultrasound findings within 2 years and the CT scan results prior to VATS were considered to indicate MHHs. Therefore, surgical resection of the hepatic masses was not performed.

Thirty-two months later, abdomen CT showed no significant changes in the hepatic masses. However, chest CT indicated recurrence of IH, and the 5th rib was slightly eroded (Fig. 1F, 1G).

## 3. Literature review

We searched the English literature relating to IH between January 1990 and September 2023 and identified 18 case reports.^[1–18]^ Our present case represents the 19th case (Table 1). By reading these reports, we found no difference in the prevalence of IH between men and women, and most were young patients without chest symptoms; 2 patients had a history of trauma; 1 patient had other vascular lesions, and more than half of the lesions originated from the intercostal muscles and some patients had rib destruction. Magnetic resonance imaging (MRI) may have advantages over CT in terms of diagnostic accuracy. Thus far, all patients underwent IH resection except for 1 patient who opted for regular observation. In addition, pathological results showed that cavernous hemangioma was the most common, followed by VH and mixed hemangioma, although there were 3 patients for whom the IH subtype was not specified.

**Table 1:** Characteristics of patients in published English literature.

Author	Age (yr), sex	Size (cm)	Intercostal space	Symptoms of chest	Rib	Pathology	Follow up	Medical history
Wincheste et al^[1]^	39, F	5.0	Right (5)	Discomfort, fullness	N	CA	–	–
Robinson ^[2]^	58 M	3.0	Right (3)	No symptoms	N	CA	–	–
Hashimoto et al^[3]^	32, M	1.2	Left (6)	No symptoms	N	VH	N (24 mo)	–
Dzian and Hamzík^[4]^	36, M	9.5	Left (7–8)	Pain	Unclear	VH	N (10 mo)	–
Dantis et al^[5]^	18, M	6.0	Left (7)	Swelling, pain	Y	CA	N (12 mo)	–
Ulku et al^[6]^	11, F	8.5	Right (9–11)	Unclear	N	CA	N (6 mo)	–
Saldanha et al^[7]^	34, F	3.0	Left (3)	Unclear	Unclear	CA	N (12 mo)	–
Elbawab etal^[8]^	14, M	6.0	Right (5)	No symptoms	Y	Hemangioma	N (6 mo)	–
Agarwal et al^[9]^	44, F	Unclear	Right (3)	No symptoms	N	Hemangioma	N (18 mo)	–
Mei et al^[10]^	14, F	7.0	Right (4)	Pain, hemoptysis	Y	Hemangioma	N (10 mo)	Trauma
Aguilo et al ^[11]^	23, F	5.0	Left (7)	No symptoms	N	AVH	–	Subdural AVM
Kara et al^[12]^	46, M	4.0	Right (2)	No symptoms	N	AVH	N (48 mo)	–
Yuan et al^[13]^	44, F	Unclear	Left (2)	No symptoms	N	CA + CH	N (36 mo)	–
Ono et al^[14]^	33, M	9.5	Right (3–5)	No symptoms	N	SVIH + LVIH	N (36 mo)	–
Ali et al^[15]^	22, F	7.0	Left (6–9)	No symptoms	Unclear	CA + CH	N (6 mo)	–
Yonehara et al^[16]^	33, M	5.0	Left (6)	No symptoms	Y	SVIH	N (60 mo)	Trauma
Kubo et al^[17]^	27, M	5.5	Right (7)	Pain, exertional dyspnea	N	LVIH	N (6 mo)	–
Ochi et al^[18]^	17, M	2.9	Left (2)	No symptoms	N	SVIH	N (18 mo)	–
Current case	58, M	1.9, 1.1	Left (5)	No symptoms	Y (after 32 mo)	VH	Y (32 mo)	MHHs

F = female, Y = yes, N = no, AVH = arteriovenous hemangioma, AVM = arteriovenous malformations, CA = cavernous hemangioma, CH = capillary hemangioma, VH = venous hemangioma, SVIH = intramuscular hemangioma of small-vessel type, LVIH = intramuscular hemangioma of large-vessel type.

## 4. Discussion and conclusions

Hemangiomas of the chest wall are very rare, especially in the intercostal region. IHs can originate from the intercostal muscles or connective tissue outside the pleura.^[10,14]^ IHs are usually considered to be congenital or due to trauma.^[5,6]^ Our patient had no history of chest trauma, but had MHHs. Hemangiomas can be divided into a variety of subtypes. At present, the most commonly used classification system is based on the type of vascular structure, which includes 5 subtypes: capillary hemangioma, cavernous hemangioma, VH, arteriovenous hemangioma, and mixed hemangioma.^[5]^ Pathology confirmed that both tumors in our patient were VHs. Although IH is a benign tumor, it is somewhat aggressive. In past cases, more than half of the patients had their ribs removed near the tumor. In our case, no obvious rib erosion was found during surgery, although recurrent IH was found to erode adjacent rib during follow-up.

Thus far, there have been no review articles on the imaging findings of IHs, and this condition is often misdiagnosed as other non-vasogenic lesions prior to surgery. Therefore, we reviewed and analyzed the previously reported CT and MRI findings of IHs, and summarized the imaging features combined with our case. By reviewing the reported literature related to plain CT images, we found that IH is a mass with a clear boundary that features a uniform or slightly uneven density and may include lobulation or phleboliths or fat density.^[6]^ phlebolith is a unique sign of hemangiomas.^[19]^ CT and MRI are both sensitive to phleboliths. In our case, phlebolith was not detected on CT images; this was the main cause of misdiagnosis. Moreover, the tumors found in our patient contained fat density; this also caused confusion with regards to hamartoma. MRI enables multidirectional, multisequence imaging of tumors with high soft tissue resolution and the analysis of tumor properties based on the signal strength displayed by different sequences. IH generally exhibits medium or slightly lower signals than adjacent muscle tissue on T1-weighted MRI,^[8,15]^ but sometimes it can also appear as a high signal intensity.^[6]^ On T2-weighted MRI, IH showed a high signal that remained high even after fat suppression.^[8]^ There are various enhanced manifestations of IH that need to be considered. CECT and MRI scans can show a similar pattern of enhancement to hepatic hemangioma, with typical “fast in, slow out” or gradual “filling” enhancement,^[8]^ and some IHs can also present with nodular/tubular enhancement and central enhancement,^[15]^ as in our case. However, due to a lack of understanding with regards to IH, we did not make a correct diagnosis. The literature shows that a few IHs may not show marked enhancement.^[3,10]^ In addition, the imaging examinations of our patient showed that MHHs were present; this may be helpful for the diagnosis of IH. Although there was only 1 IH coexisting with subdural hemangioma in the previous literature, which was similar to our case, we believe that this condition may require the attention of diagnostic physicians.

Early clinical intervention for IHs can prevent tumor growth and damage to adjacent structures, such as the bone. Imaging, especially MRI, is helpful for the diagnosis of IH and the formulation of surgical protocols. According to literature review, the preoperative biopsy of tumors suspected of hemangioma is not recommended because of the risk of massive bleeding and the possibility that biopsy results may remain inconclusive.^[17]^ Surgical resection is the most common and optimal method for the treatment of IH. Furthermore, it is important to note that if there is a large blood vessel supplying the tumor, vascular embolization or ligation is recommended before resection.^[8]^ In addition, due to the potential for recurrence or regeneration of the residual tissue, we recommend that not only complete resection of the IH should be performed during surgery, but also intraoperative pathological examination should be performed to ensure that the submicroscopic margin is negative.

## Acknowledgments

We would also like to thank the Director of the Pathology, Department of Ziyang People’s Hospital for his contributions to this research.

## Author contributions

**Investigation:** Xiangui Li, Xing Wen.

**Writing – original draft:** Xiangui Li, Lin Yang.

**Writing – review & editing:** Xing Wen, Lin Yang.
